# Reply to: “Understanding virologic heterogeneity in chronic hepatitis B treatment”

**DOI:** 10.1016/j.jhepr.2024.101280

**Published:** 2024-11-29

**Authors:** Philippa C. Matthews, Tingyan Wang, Eleanor Barnes, the Health Informatics Collaborative for Viral Hepatitis

**Affiliations:** 1 The Francis Crick Institute, 1 Midland Road, London NW1 1AT, UK; 2 Division of Infection and Immunity, University College London, Gower Street, London, WC1E 6BT, UK; 3 Department of Infectious Diseases, University College London Hospital, Euston Road, London NW1 2BU, UK; 4 Mortimer Market Centre, Central North West London NHS Trust, Capper Street, London WC1E 6JB, UK; 5 NIHR Oxford Biomedical Research Centre, Oxford, UK; 6 Nuffield Department of Medicine, University of Oxford, Oxford, UK; 7 NIHR Health Informatics Collaborative, Oxford University Hospitals NHS Foundation Trust, Oxford, UK; 8 Department of Hepatology, Oxford University Hospitals NHS Foundation Trust, Oxford, UK

To the Editor:

We thank Dr Luo and colleagues for their letter^1^ in response to our recent analysis from the UK NIHR Health Informatics Collaborative (HIC) for Viral Hepatitis. We reported our analysis of a large longitudinal real-world population, in which we classified responses to nucleos(t)ide analogue (NA) therapy in HBV infection.^2^ Herein, we address three specific questions raised by Luo *et al.*

***1***. ***What clinical, imaging and laboratory markers should be explored to determine HBV treatment outcome?***

We selected viral load (VL) as a biomarker for longitudinal analysis, as all current clinical practice guidelines recommend routine measurement (*e.g.*^3^^,^^4^), reflecting the established position of VL as a risk factor for liver disease in HBV infection, together with its significance in assessment of transmission risk. Furthermore, VL is the correct biological outcome marker of NA efficacy as these agents should suppress the generation of new viral DNA genomes (quantified in the peripheral circulation) from RNA templates in infected hepatocytes.

However, surveillance and risk stratification must also incorporate the long-term clinical impact of HBV therapy. On these grounds, rather than defining VL as a primary outcome maker, we undertook multivariate analysis to evaluate to what extent VL trajectory is associated with liver disease progression.^2^ On this basis, we reported that individuals in our ‘slow virological suppression’ group had approximately 2-fold increased hazards of progression to fibrosis/cirrhosis compared to those with suppressed viraemia based on a range of clinical assessment tools.

Given the progressive focus on HBV functional cure, there is interest in quantification of HBV surface antigen (HBsAg).^5^ However, there are currently several challenges in incorporating results of this assay into analysis of real-world cohorts. First, quantitative HBsAg (qHBsAg) measurement has not been a routine part of risk assessment or clinical monitoring to date, and many settings do not offer an assay. Thus, qHBsAg readings were available for <20% of individuals in our dataset, and <15% on a longitudinal basis. Second, even when available, measurement is not yet consistent; assays variably generate fully quantitative or semi-quantitative read-outs, making it difficult to pool data from different settings.^6^ Finally, data interpretation is complex, as qHBsAg reflects not only intrahepatic transcriptional activity, but also translation of viral DNA that has been integrated into the host genome.

Our original qHBsAg analysis (using the available data) showed a decrease over time in classes with VL suppression.^2^ We agree that enhanced data collection and analysis will be important for future research, and will become increasingly tractable as access to standardised laboratory assays for HBsAg quantification becomes more routine.

**2**. **How can we assess clinical interventions in the context of ‘slow virologic suppression’ on NA therapy?**

Interrogation of the outcomes of specific interventions with sub-groups is beyond the scope of the original study, and is limited by small numbers in the slow suppression group and heterogeneity in clinical practice. Interventions might include use of different agents or drug combinations. However, to date, tenofovir alafenamide (TAF) has not been accessible in the UK as monotherapy for HBV and can only be accessed by drawing on combination therapy for HIV treatment and prophylaxis.

As the HIC dataset expands, we will gain power, and further analyses will include the impact of switching agent or adding a second agent. Our consortium is currently working on an analysis of dual therapy; preliminary data were presented at the EASL meeting in 2024.^7^

**3**. **Do patients remain in fixed trajectory classes throughout treatment?**

The mathematical model we applied is agnostic to any *a priori* assignment of VL class, simply assigning each individual into a category that best describes the longitudinal pattern observed within the period of observation.^2^ This makes the model flexible and responsive to different dynamics, accounting for significant heterogeneity. It is already clear that individuals classified into the ‘slow suppression’ group are likely to achieve aviraemia with prolonged treatment. Conversely, we recognise that some individuals classified by our model into the ‘long-term suppression’ group (Class I) may have achieved this endpoint over a prolonged period prior to cohort entry, and if earlier data had been available they might have been assigned into a different class.

We agree that recognising slow suppression as a treatment phenotype is of clinical importance, for example in considering when further interventions might be warranted, and/or in assessing virologic suppression in the context of perinatal prophylaxis (where NA outcomes are time-sensitive).^8^ Given the HIC approach to collating multi-centre longitudinal data, we can take an extended view over time as the cohort expands and explore the impact of new treatments as they emerge.

In conclusion, we are grateful for the engagement in determining the potential of this expanding real-world dataset. Such dialogue will ensure these data are optimally used to inform an understanding of risk stratification, and to refine clinical management strategies over time as more people living with HBV become treatment eligible.

## Financial support

This work has been conducted using 10.13039/501100000272National Institute for Health and Care Research 10.13039/100018696Health Informatics Collaborative (10.13039/501100000272NIHR HIC) data resources and funded by the 10.13039/501100000272NIHR HIC. E.B. is an 10.13039/501100000272NIHR senior investigator. P.C.M. is funded by the 10.13039/100010438Francis Crick Institute (ref CC2223), and UCLH 10.13039/501100000272NIHR Biomedical Research Centre. The views expressed in this article are those of the authors and not necessarily those of the National Health Service, the NIHR or the Department of Health.

## Authors' contributions

The primary version of this letter was drafted by PCM with comments and edits from TW and EB. All authors reviewed and agreed the final text.

## Conflict of interest

EB holds a collaborative research grant from GSK using data from the NIHR health informative initiative and Vaccitech/Barinthus research grant in HBV vaccines, and declares License fees from Vaccitech for HBV and HCV vaccine development, Vaccitech honoraria for conference presentation, and patents in HBV and HCV vaccine antigens. PCM declares Funding from GSK to support a PhD fellowship in her programme (2019-2022.).

Please refer to the accompanying ICMJE disclosure forms for further details.

## References

[1] Luo R., Ran X., Sun R. (2024). Understanding virologic heterogeneity in chronic hepatitis B treatment. J Hepatol Rep.

[2] Wang T., Campbell C., Stockdale A.J. (2024). Distinct virologic trajectories in chronic hepatitis B identify heterogeneity in response to nucleos/tide analogue therapy. JHEP Rep.

[3] European Association for the Study of the Liver (2017). EASL 2017 Clinical Practice Guidelines on the management of hepatitis B virus infection. J Hepatol.

[4] (2024). Guidelines for the prevention, diagnosis, care and treatment for people with chronic hepatitis B infection.

[5] Höner Zu Siederdissen C., Maasoumy B., Cornberg M. (2017). What is new on HBsAg and other diagnostic markers in HBV infection?. Best Pract Res Clin Gastroenterol.

[6] Wang T., Smith D.A., Campbell C. (2023). Cohort profile: the national Institute for health research health Informatics collaborative: hepatitis B virus (NIHR HIC HBV) research dataset. Int J Epidemiol.

[7] Im Y.R., Tan C., Wang T. (2024). Retrospective analysis of longitudinal data for UK adults living with chronic hepatitis B virus (HBV): characteristics of the population on dual therapy.

[8] Matthews P.C., Ocama P., Wang S. (2023). Enhancing interventions for prevention-of-mother-to-child- transmission (PMTCT) of hepatitis B virus (HBV). JHEP Rep.

